# Editorial: Extracts from plants and other natural sources: application, characterization, optimization, and their use, volume II

**DOI:** 10.3389/fnut.2026.1919967

**Published:** 2026-08-21

**Authors:** Saša D. Ðurović, Yulia A. Smyatskaya, Yuri V. Shatalin

**Affiliations:** 1 Institute of General and Physical Chemistry, Belgrade, Serbia; 2 Graduate School of Biotechnology and Food Industries, Peter the Great Saint-Petersburg Polytechnic University, Saint Petersburg, Russia; 3 Institute of Theoretical and Experimental Biophysics, Russian Academy of Sciences, Pushchino, Russia

**Keywords:** application, characterization, extraction, extracts, natural compounds, optimization

Natural compounds still draw significant attention from the scientific community. Their diverse applications have led to numerous publications that study their isolation, biological activity, and potential applications across different fields and industries. To address the extensive interest of the scientific community, we launched this Research Topic titled “*Extracts From Plants and Other Natural Sources: Application, Characterization, Optimization, and Their Use, Volume II*,” the continuation of our Research Topic dedicated to this significant scientific field.

This Research Topic, with its 15 publications, focuses more on the biological activity of various natural materials, their extracts, and specific compounds. Castro-Saavedra et al. studied depigmenting and tyrosinase inhibition potential of alkaloids isolated from Chilean flora (*Cryptocarya alba, Peumus boldus*, and *Laurelia sempervirens*) using experiments and *in silico* methods (molecular docking). Abdul-Hafeez et al. investigated the composition of *Vachellia nilotica* fruit extract for its cancer activity. The authors reported the presence of several classes of natural compounds (such as terpenoids, flavonoids, tannins, and alkaloids) which exhibit strong antioxidant activity and moderate, selective cytotoxicity. Sousa de Sa et al. used different analytical techniques (spectrophotometry and HPLC) to characterize *Cannabis sativa* root. Furthermore, pharmacological effects and safety have also been studied and reported using different *in vitro* assays and models reporting quite promising results. Liu, Zhou et al. also studied the roots of the Chinese traditional plant *Gardenia jasminoides*. The authors combined various analytical tools, biological assays, and *in silico* techniques to identify the compounds responsible for the biological activity (seven compounds have been identified and reported). Song et al. reported a procedure for isolating exosome-like nanovesicles from decoction made of Yiqi Huoxue Jiedu and tested them for anti-ovarian cancer activity. Furthermore, Wu et al. investigated the activity of *Panax notoginseng* exosomes on mesenchymal stem cells derived from rat's bone marrow. Authors also reported data on signaling mechanisms inside the studied cells.

Saccharides are also one of the most extensively studied classes of compounds. Chen et al. reported on the extraction, purification, and characterization of polysaccharides from *Dendrobium findlayanum*. The authors also studied biological activities and potential therapeutic applications of isolated polysaccharides. They reported huge application potential as a nutraceutical supplement to relieve inflammation of the intestine caused by the oxidative stress. Lin et al. reported on the application of UHPLC-Q-TOF-MS/MS for structural elucidation of glycosidic resins isolated from sweet potato cultivars and studied their lipase inhibitory activity.

Plants generally exhibit a broad spectrum of biological activities and are used in both the pharmaceutical and food industries. Thus, Pan et al. provided a significant review on the botany, traditional application, processing, toxicity, phytochemistry, and pharmacology of *Rhizoma alismatis*. The authors indicated that their studies have demonstrated diuretic, liver-protective, antihypertensive, antihyperglycemic, anticancer, anti-inflammatory, and antioxidant activities. Liu, Jia et al. also provided a review on application, anti-tumor activity, action mechanisms, and preventive healthcare of *Platycodon grandiflorum*. Based on the available studies, the authors reported that phenolic acids, flavonoids, and polysaccharides are main classes of natural compounds responsible for reported activity. However, plants are not the only source of valuable compounds and different classes. Yang et al. provided a review of the biosynthesis, technological advancements, and practical applications of lipids derived from marine microalgae.

In addition to different classes of natural compounds, certain articles aimed to study specific compounds. Thus, Liu, Lei et al. reported on properties, synthesis, mechanism of action, and applications of thymol, a major compound in thyme essential oil. Liu, Pan et al. studied parishin E isolated from ginger-processed *Gastrodia elata* Bl (prepared by stir-frying GEB moistened with ginger juice). The authors concluded that parishin E relieves rheumatoid arthritis throughout the regulation of lactylation of histone 3 at H3K18la and H3K27la sites and marked it as a promising drug for rheumatoid arthritis.

However, studying the biological activity of compounds and extracts derived from various natural sources is not the only goal. There are several directions of research in this field. Among them are the development of analytical methods and protocols and the optimization of the processes. Yan et al. developed a TaqMan qPCR method to identify and quantify *Fritillaria hupehensis* and specifically distinguish it from *Fritillaria thunbergii* Miq. Last but not least, Micić et al. optimized the microwave-assisted extraction of summer savory using an artificial neural network. They studied the kinetics of extraction process, chemical profile, and thermal properties of the extracts.

The number and quality of the published articles in Volume II of this Research Topic showed that the field remains highly impactful on both the scientific community and industry. Volume II continued the mission of this Research Topic: to fill gaps and expand knowledge in this field. This Research Topic continues to provide significant information about the natural compounds, their behavior, and biological activity.

